# Effect of Vapor-Phase Oregano Essential Oil on Resistant *Candida* Species Biofilms: Mechanisms of Action

**DOI:** 10.1128/spectrum.05124-22

**Published:** 2023-03-27

**Authors:** Liliana Fernandes, Raquel Costa, Sónia Silva, Mariana Henriques, Sofia Costa-de-Oliveira, Maria E. Rodrigues

**Affiliations:** a Centre of Biological Engineering, LMaS—Laboratório de Microbiologia Aplicada à Saúde, University of Minho, Campus de Gualtar, Braga, Portugal; b LABBELS—Associate Laboratory, Braga/Guimarães, Portugal; c Aromas Aqua Spa—Clínica Saúde, Vila Verde, Braga, Portugal; d National Institute for Agrarian and Veterinary Research, Vairão, Vila do Conde, Portugal; e Division of Microbiology, Department of Pathology, Faculty of Medicine, University of Porto, Porto, Portugal; f Center for Health Technology and Services Research—CINTESIS@RISE, Faculty of Medicine, University of Porto, Porto, Portugal; University of Guelph

**Keywords:** vulvovaginal candidiasis, resistant *Candida* species, phytotherapeutic applications, oregano essential oil, vapor phase of essential oil, mechanisms of action

## Abstract

Vulvovaginal candidiasis (VVC) is one of the most prevalent vaginal infectious diseases. The increasing incidence of drug-resistant *Candida* strains and the limited therapeutic options make the discovery of effective alternative therapies fundamental. Essential oils (EOs) have been suggested as a promising alternative, and interestingly, vapor-phase essential oils (VP-EOs) present more advantages than their direct application. Thus, this study aims to evaluate the effect of oregano VP-EO (VP-OEO) on biofilms of antifungal-resistant vaginal isolates of *Candida* species (Candida albicans and Candida glabrata) and determine its mode of action. CFU, membrane integrity, and metabolic activity were evaluated. Furthermore, a reconstituted vaginal epithelium was used to mimic vaginal conditions and evaluate the effect of VP-OEO on *Candida* species infection, analyzed by DNA quantification, microscopy, and lactate dehydrogenase activity. The results revealed high VP-OEO antifungal activity. There was a significant reduction (>4 log CFU) in *Candida* species biofilms. Furthermore, the results show that the mechanisms of action of VP-OEO are related to membrane integrity and metabolic activity. The epithelium model confirms the effectiveness of VP-OEO. This study suggests that VP-EO can be considered a first approach for the development of an alternative form of VVC treatment.

**IMPORTANCE** This work presents a new approach to the application of essential oils, exposure to the vapor phase, which can be considered a first approach for the development of a complementary or alternative form of vulvovaginal candidiasis (VVC) treatment. VVC is a significant infection caused by *Candida* species and remains a common disease that affects millions of women every year. The great difficulty in treating VVC and the extremely limited effective therapeutic options make the development of alternative treatments crucial. In this scope, this study aims to contribute to the development of effective, inexpensive, and nontoxic strategies for the prevention and treatment of this infectious disease, based on natural products. Moreover, this new approach has several advantages for women, such as lower costs, easy access, an easier mode of application, avoidance of skin contact, and, therefore, fewer negative impacts on women’s health.

## INTRODUCTION

Antimicrobial resistance is increasing over time, threatening the effective prevention and treatment of an ever-increasing range of infectious diseases (1). One of the most common and frequent fungal infections is candidiasis, ranging from superficial mucosal infections (oral cavity, vagina, penis, gastrointestinal tract, or other parts) to systemic and potentially fatal diseases (disseminated candidiasis) (2). Indeed, vulvovaginal candidiasis (VVC) affects millions of women of reproductive age every year, causing physical discomfort, pain, and mental distress and representing considerable direct and indirect costs (3, 4). VVC is caused by *Candida* species, namely, Candida albicans and Candida glabrata, both phenotypically and genetically different (5). Although C. albicans is more frequently associated with candidiasis, the incidence of infections caused by C. glabrata is increasing, showing overall greater resistance to common antifungal agents, especially azoles (6–8). These species are associated with biofilm formation, one of the main virulence factors associated with increased resistance to antifungal agents (6, 7). Even though several years ago, antifungal agents exerted a potent effect, these agents are currently no longer effective (9). Thus, discovering new and effective alternative therapies or improving/reinventing already used therapies is fundamental.

From the past until today, plants and their derivatives have been employed in traditional medicine, alternative medicine, or phytotherapeutic applications for the prevention and treatment of several diseases (10). In this sense, essential oils (EOs) have been suggested as potential sources of new therapeutic products due to their antileishmanial, antiviral, anti-inflammatory, antioxidant, hepatoprotective, antitumor, and antimicrobial activities (11, 12). Furthermore, EOs have advantages such as fewer side effects, less toxicity, and better biodegradability than available antimicrobial agents (13). These natural products are biosynthesized by glandular trichomes and other secretory structures in plants, being liquids that are particularly rich in volatile molecules such as monoterpenes and sesquiterpenes with their oxygenated derivatives (phenols, oxides, esters, ketones, and aldehydes), phenylpropanoids (alcohols, aldehydes, phenols, and short-chain aliphatic hydrocarbon), and nitrogenous or sulfured components (14, 15). The inhibitory activities of EOs against many fungal pathogens have been widely reported (16), including EOs from the Lamiaceae family such as *Origanum* species and *Thymus* species, which have been described as being particularly effective against various microorganisms, particularly *Candida* species (17–22). In fact, oregano EO (OEO) was able to inhibit, *in vitro*, the germination and development of the filamentous form of C. albicans (8, 17, 23). Moreover, Hacioglu et al. (24) also showed previously that OEO inhibits the three phases of *Candida* biofilms, i.e., their adhesion, formation, and mature state (24). Those authors also showed the ability to reduce biofilm formation by up to 50% when biofilms were formed on surfaces previously coated with OEO (24).

The literature provides information about the impact of the direct application of EOs on *Candida* species (10, 11, 16), but few data are available on the antifungal effect of the vapor-phase of essential oils (VP-EOs) (25), mainly on *Candida* species biofilms. Despite this, studies have shown that VP-EO showed greater antimicrobial activity than its liquid phase (26, 27).

The objective of this work was to evaluate the abilities of the vapor-phase of oregano essential oil (VP-OEO) to inhibit biofilm formation and destroy mature biofilms of antifungal-resistant vaginal isolates of *Candida* species. Furthermore, the mode of action of VP-OEO against two antifungal-resistant vaginal isolates of *Candida* species (C. albicans and C. glabrata) was evaluated.

## RESULTS AND DISCUSSION

The high incidence of resistant *Candida* species and the associated negative consequences and therapeutic limitations make it crucial to develop effective, inexpensive, and nontoxic alternative treatments. In this sense, OEO has demonstrated good fungicidal and bactericidal activities against different pathogens (28–30). Recent studies also showed that the volatile part of the EO (VP-OEO) has interesting antimicrobial activity (31–33). Thus, considering the highly effective anti-*Candida* activity evidenced by OEO described in the literature, it is essential to deepen the knowledge of the effect of the vapor-phase of these oils alone, a phase that is often neglected.

**FIG 1:**
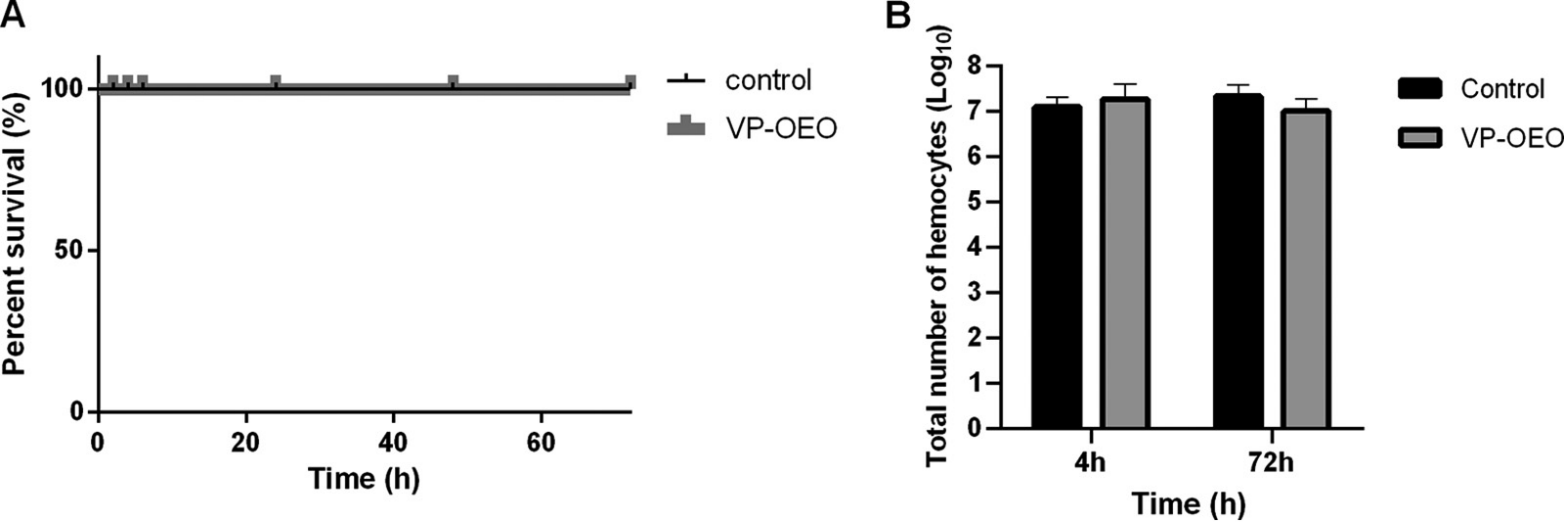
Toxicity of the vapor-phase of oregano essential oil (VP-OEO) measured in a Galleria mellonella model. (A) Survival curves of G. mellonella larvae exposed to VP-OEO and the respective controls (without exposure to VP-OEO). (B) Total numbers of hemocytes counted in G. mellonella larvae after 4 and 72 h of exposure and those unexposed to VP-OEO.

In the first stage of this study, the *in vivo* toxicity of VP-OEO was evaluated using the Galleria mellonella model (34). After 72 h of exposure of larvae to VP-OEO, the results revealed that was no evidence of toxicity. In fact, until 72 h, all larvae remained alive (Fig. 1A), with no significant differences in the total numbers of hemocytes in the larval hemolymphs (Fig. 1B) exposed and not exposed to VP-OEO.

After verifying the nontoxicity of VP-OEO, the antifungal activity of VP-OEO against antifungal-resistant vaginal isolates of several *Candida* species was evaluated, and the effect of VP-OEO was compared with that of direct contact with OEO (DC-OEO). For this, an initial screening of the OEO against antifungal-resistant vaginal isolates of C. albicans, C. glabrata, C. guilliermondii, and C. krusei was performed using the disk diffusion method (DC and VP) (Table 1).

**TABLE 1:** Anti-*Candida* activities of direct-contact and vapor-phase oregano essential oils against drug-resistant isolates evaluated using the disk diffusion method^*a*^

Isolate	Mean oregano essential oil inhibition zone (mm) ± SD	
	Direct contact	Vapor-phase
C. glabrata		
Cg1	52.7 ± 2.5	54.7 ± 2.1
Cg2	51.0 ± 5.6	57.3 ± 2.5
Cg3	38.0 ± 2.8	31.3 ± 2.3
Cg4	68.0 ± 3.5	56.0 ± 5.3***
Cg5	51.7 ± 2.9	50.0 ± 3.0
Cg6	57.7 ± 4.0	44.0 ± 3.6***
Cg7	43.0 ± 2.5	36.0 ± 3.6
Cg8	38.3 ± 1.2	36.3 ± 2.3
C. albicans		
Ca1	53.7 ± 3.2	30.3 ± 2.9****
Ca2	46.3 ± 4.2	35.3 ± 3.8*
Ca3	53.3 ± 6.1	27.5 ± 2.1****

C. krusei Ck1	50.8 ± 2.2	43.7 ± 3.2*
C. guilliermondii Cgi1	62.0 ± 2.7	77.3 ± 3.1***

a Asterisks indicate statistical significance between direct-contact oregano essential oil (DC-OEO) and vapor-phase OEO (VP-OEO) (*, *P* < 0.1; **, *P* < 0.01; ***, *P* < 0.001; ****, *P* < 0.0001).

Both DC-OEO and VP-OEO were able to inhibit the growth of all tested isolates but with different impacts (inhibition zones of 27 to 77 mm), and different results were obtained depending on the *Candida* species. The effects of DC-OEO and VP-OEO were similar in almost all of the C. glabrata isolates except the C. glabrata Cg4 and C. glabrata Cg6. In these two strains, VP-OEO (*P* < 0.001) had a smaller effect than DC-OEO. In contrast, significantly reduced effects on C. albicans and C. krusei were obtained with VP-OEO (*P* < 0.1 and *P* < 0.0001, respectively), and the opposite was observed for C. guilliermondii (*P* < 0.001). In fact, C. guilliermondii proved to be more susceptible to both DC-OEO and VP-OEO. The different antifungal activities of DC-OEO and VP-OEO may result from the characteristics of the EOs. Indeed, in the liquid phase (DC-EO), the activity of the EOs depends on two factors, the diffusibility and solubility of the EOs in the medium, while the effect of VP-EO depends only on the volatility of the EO (27). For the study of the mechanism of action of EOs or volatile substances, volatility and hydrophilicity or lipophilicity must be factors to be considered. Indeed, the lipophilic character allows interaction with lipid structures of the fungal cell membrane, allowing easy penetration and modifying cell membrane properties such as permeability and fluidity. Consequently, fungal cells suffer structural damage that can result in cell death (32). However, the lipophilic molecules in the aqueous medium associate to form micelles, suppressing the fixation of EOs on the microorganism, while in the VP-EO, it allows free fixation (33). The level of lipophilicity is related to the polar functional groups in its constitution (35).

According to these data, it can be said that the results obtained during this work are closely related to the composition of the OEO (Table 2). This EO contained the following major components: carvacrol (36.97%, vol/vol), thymol (21.44%, vol/vol), *p*-cymene (15.27%, vol/vol), and γ-terpinene (13.35%, vol/vol). However, several factors must be considered to predict the volatility of compounds, such as molar mass, vapor pressure, molecular structure (the existence of functional groups), log *K_ow_* (octanol/water partition coefficient), and polarity.

**TABLE 2:** Compounds identified in oregano essential oil and their characteristics such as vapor pressure, water solubility, and log *K_ow_*

Compound, molecular formula (molar mass [g/mol])	% in oregano oil	Molecular structure	Vapor pressure (mm Hg) (at 25°C)	Solubility in water (mg/L) (at 25°C), solubility category	Log *K_ow_*
Carvacrol, C_10_H_14_O (150.217)	36.97	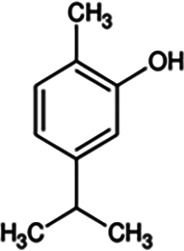	0.029	1,250, high	3.49
Thymol, C_10_H_14_O (150.22)	21.44	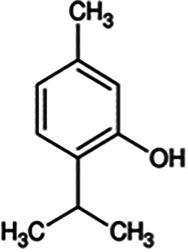	0.038	900, moderate	3.30
*p*-Cymene, C_10_H_14_ (134.21)	15.27	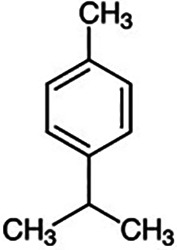	1.50	23.4, moderate	4.10
γ-Terpinene, C_10_H_16_ (136.23)	13.35	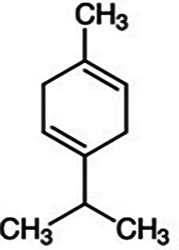	1.1	8.68, low	4.36
α-Terpinene, C_10_H_16_ (136.23)	2.04	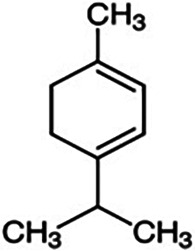	1.66	5.915, low	4.53
α-Caryophyllene, C_15_H_24_	1.97	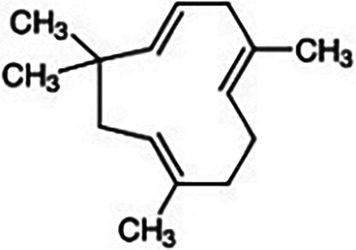	0.008	0.014, low	6.59
Linalool, C_10_H_18_O (204.35)	1.55	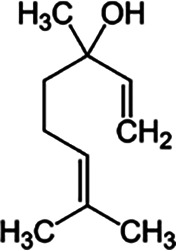	0.16	1,590, high	2.97
Myrcene, C_10_H_16_ (136.23)	1.39	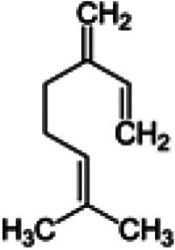	2.29	5.6, low	4.17
α-Thujene, C_10_H_16_ (136.238)	0.89	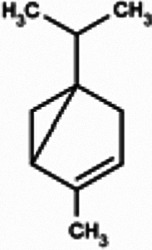	4.77	2.91, low	4.02
α-Pinene, C_10_H_16_ (136.24)	0.66	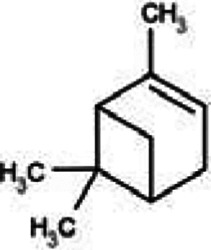	4.75	2.49, low	4.83
Terpinen-4-ol, C_10_H_18_O (154.25)	0.63	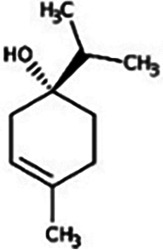	0.048	386.6, moderate	3.26
Limonene, C_10_H_16_ (136.24)	0.55	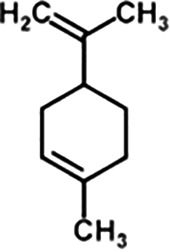	1.54	4.58, low	4.38

*p*-Cymene, γ-terpinene, α-terpinene, myrcene, α-thujene, α-pinene, and limonene are the most volatile components (vapor pressure of >1). As monoterpenes (such as γ-terpinene, α-terpinene, myrcene, α-thujene, α-pinene, and limonene) do not have functional groups, these compounds have the highest vapor pressure and therefore are the most volatile and also the most lipophilic (higher log *K_ow_*). Indeed, Muñoz et al. (36) demonstrated previously that limonene acts on yeast genetic material, leading to damage to the cell wall and intracellular structures, including nuclear alterations, and induces dramatic structural changes in organelles, accompanied by cell wall disruption. Of the monoterpene compounds, the cyclic monoterpenes α-thujene and α-pinene are the most volatile (4.75 to 4.77 mm Hg of vapor pressure), although they are present at a lower percentage of the total oil composition. Nóbrega et al. (37) showed previously that α-pinene showed significant antifungal activity against *Candida* isolates and fungicidal action and proved to be effective in inhibiting fungal structures such as pseudohyphae and promoting a marked decrease in blastoconidia. Linalool and terpinen-4-ol are also monoterpenes but with alcohol function, so these compounds are more hydrophilic.

In turn, *p*-cymene, carvacrol, and thymol are aromatic compounds with a benzene ring; however, *p*-cymene, as it is not a phenol, unlike carvacrol and thymol, which have the hydroxyl group attached directly to the benzene ring, becomes less volatile and hydrophilic. Several studies have shown that carvacrol (high solubility) and thymol (moderate solubility) exerted interesting anti-*Candida in vitro* activity as determined by broth microdilution (DC), even against fluconazole-resistant Candida tropicalis and C. glabrata strains (38–40). Indeed, high concentrations of carvacrol may be directly related to germ tube inhibition and are also often considered mainly responsible for modifying membrane permeability (23, 41–43). However, studies that compared the antimicrobial effects of EOs and purified components, such as carvacrol, in equal concentrations showed that EOs were more effective than the purified compounds (44). Thus, the synergism between carvacrol and other components at lower concentrations may be more effective than purified carvacrol (23). In fact, carvacrol together with thymol can facilitate the entry of EO compounds through the fatty acid chains that make up the lipid bilayers of the membrane and, consequently, modify the permeability and fluidity of cell membranes (45).

**FIG 2:**
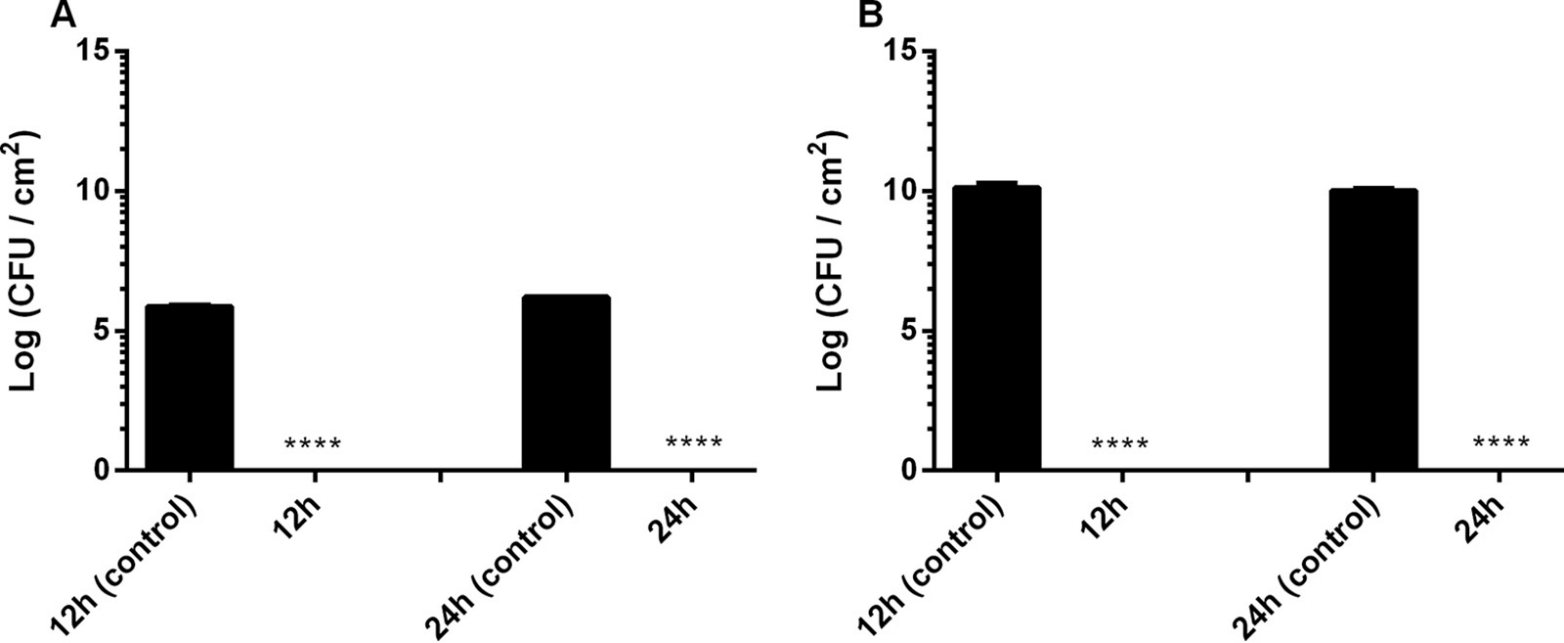
Effect of the vapor-phase of oregano essential oil (VP-OEO) on biofilm formation and preformed biofilms of the antifungal-resistant *Candida* isolates C. albicans Ca2 (A) and C. glabrata Cg8 (B). Asterisks indicate a statistically significant reduction in biofilm cell cultivability in comparison with the respective controls (*, *P* < 0.1; **, *P* < 0.01; ***, *P* < 0.001; ****, *P* < 0.0001).

Deep fungal infections, caused mainly by *Candida* species, are associated with biofilm formation that leads to the development of resistance and, consequently, become extremely difficult to treat with conventional antifungal agents (46–48). Therefore, the effects of the VP-OEO on *Candida* biofilm formation and preformed biofilms of two antifungal-resistant strains were evaluated. In this sense, vaginal isolates of C. albicans and C. glabrata were selected: C. albicans Ca2 presents extremely high-level resistance to fluconazole, and C. glabrata Cg8 presents resistance to fluconazole and ketoconazole, a less common azole (31). Thus, the results obtained from this assay showed that VP-OEO induced the total inhibition of these two species under the two conditions tested, that is, biofilm formation and preformed biofilms (Fig. 2). Exposure to VP-OEO for 24 h led to reductions of approximately 6 log CFU/mL and 10 log CFU/mL (*P* < 0.0001) for C. albicans Ca2 and C. glabrata Cg8, respectively. Thus, these results showed the great effectiveness of VP-OEO against both the establishment of a biofilm and mature biofilms.

These results are supported by our previous study where the antifungal activity of tea tree VP-EO (VP-TTEO) was evaluated: VP-TTEO induced a significant inhibitory effect on biofilm formation (2- to 3.5-log reductions) and caused a significant reduction in mature biofilms (3- to 5-log reductions) (31). In fact, it has been shown that due to their diffusibility and mode of contact, EOs can be effective in controlling biofilm cultures compared to planktonic cells (27). Furthermore, Karpanen et al. (49) noted previously that thymol showed increased activity against Staphylococcus epidermidis cells growing in biofilms compared to planktonic cells; thus, those authors suggested that thymol, being a phenolic compound, has hydrophilic and hydrophobic properties that may increase the diffusion of this compound in a biofilm and allow its access to fungal cells where it alters the permeability of the plasma membranes. In this study, although thymol has a low vapor pressure value (0.038 mm Hg), it is one of the main constituents of OEO, so it can be suggested that the observed effect of VP-OEO on the C. albicans Ca2 and C. glabrata Cg8 biofilms is due, in part, to this compound.

The mechanisms of action of EOs in their liquid phase have been described in the literature by several authors (32, 41). However, the antimicrobial activity mediated by the VP-EO is often neglected, underestimated, or ignored, so there are few studies focusing on the mechanisms of action of the VP-EO alone. A recognized and important fact is that the mechanism of action of the EOs can diverge according to the way in which the EOs come into contact with microorganisms; therefore, it is crucial to try to understand the mode of action involved in the effect of VP-EO. For this, assessments of cell damage and metabolic activity with VP-OEO were performed by flow cytometry and confocal laser scanning microscopy (CLSM) analyses.

**FIG 3:**
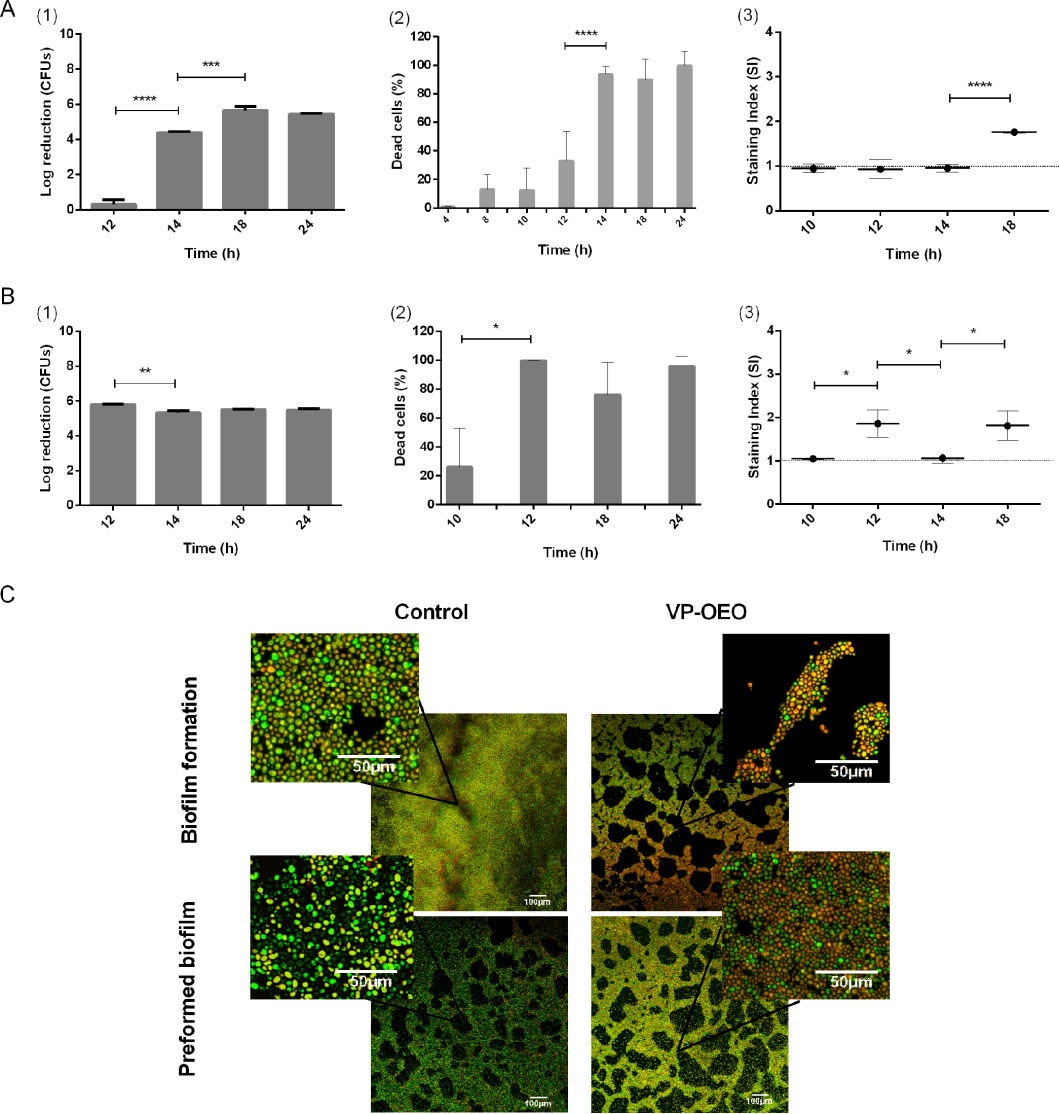
(A and B) Effects of the vapor-phase of oregano essential oil (VP-OEO) on biofilm formation (A) and preformed biofilms (B) of C. albicans Ca2, evaluated by a time-killing assay (1), cell viability (2), and metabolic activity (an SI of <1 indicates metabolic activity) (3). (C) Confocal laser scanning microscopy images of C. albicans Ca2 biofilms (biofilm formation and preformed biofilms) after 12 h of exposure to VP-OEO. CLSM images show the staining patterns for live cells (SYTO-9 [green]) and dead cells (propidium iodide [red]). Asterisks indicate a statistically significant reduction in biofilm cell cultivability compared to that at the previous time point (*, *P* < 0.1; **, *P* < 0.01; ***, *P* < 0.001; ****, *P* < 0.0001).

**FIG 4:**
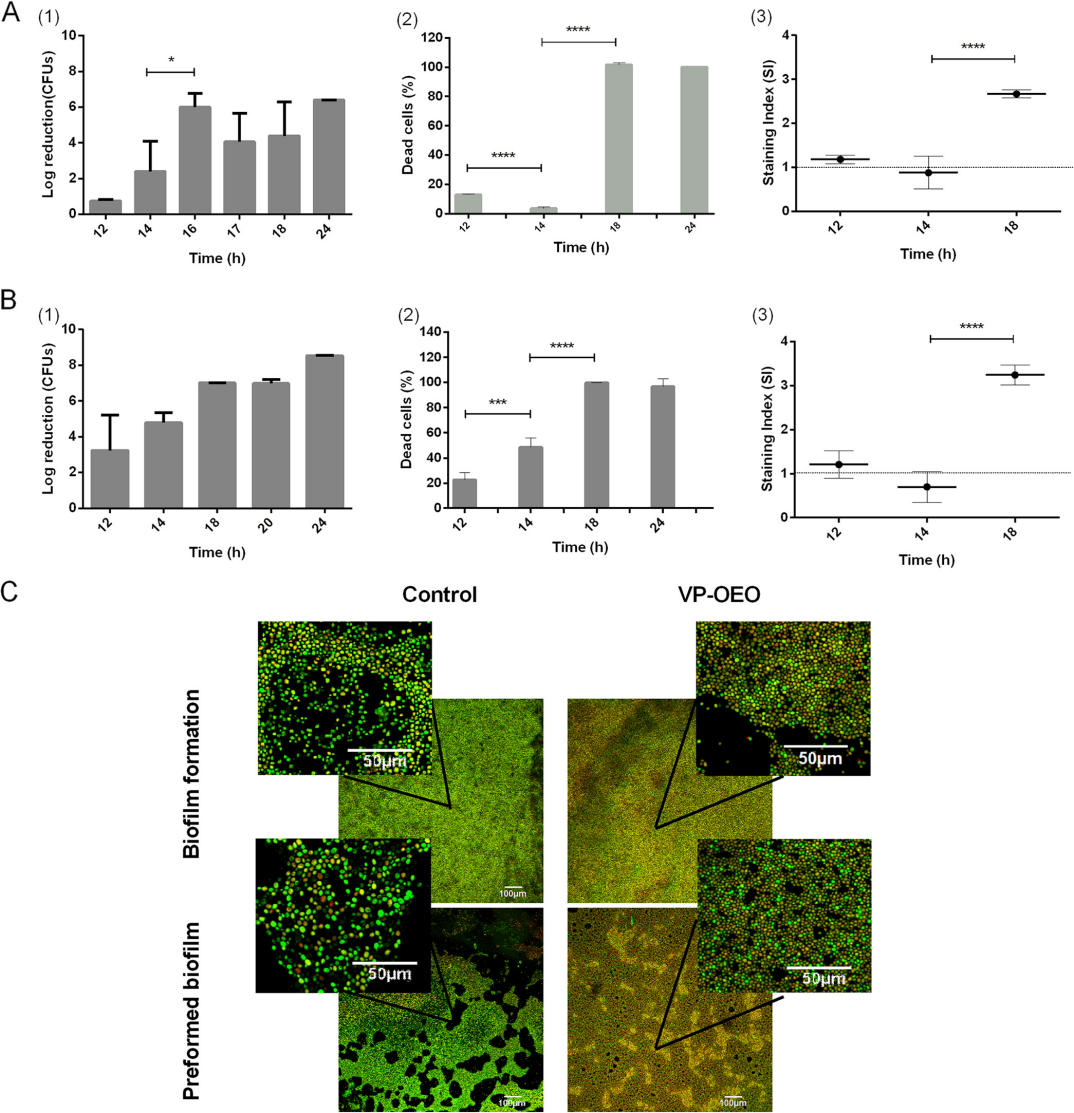
(A and B) Effects of the vapor-phase of oregano essential oil (VP-OEO) on biofilm formation (A) and preformed biofilms (B) of C. glabrata Cg8, evaluated by a time-killing assay (1), cell viability (2), and metabolic activity (an SI of <1 indicates metabolic activity) (3). (C) Confocal laser scanning microscopy images of C. glabrata Cg8 biofilms (biofilm formation and preformed biofilms) after 12 h of exposure to VP-OEO. CLSM images show the staining patterns for live cells (SYTO-9 [green]) and dead cells (propidium iodide [red]). Asterisks indicate a statistically significant reduction in biofilm cell cultivability compared to that at the previous time point (*, *P* < 0.1; **, *P* < 0.01; ***, *P* < 0.001; ****, *P* < 0.0001).

Flow cytometry has been used to determine the quantitative and qualitative properties of cells isolated from biofilms. Thus, the effect of VP-OEO was based on the detection of increased membrane permeability of *Candida* cells to propidium iodide (PI) (percentage of dead cells) and the measurement of metabolic activity using the dye 2-chloro-4-[2,3-dihydro-3-methyl-(benzo-1,3-thiazol-2-yl)-methylidene]phenylquinolinium iodide (FUN-1) (staining index [SI]), from the moment when VP-OEO started to exert an effect on cell viability according to a time-dependent killing assay (Fig. 3 and 4).

The time-dependent killing assay is used to study the activity of an antimicrobial agent and can determine the fungicidal (>3 log_10_ CFU/mL) or fungistatic (<3 log_10_ CFU/mL) activity of an agent over time (50). Thus, from this assay, it was found that after 10 to 14 h of exposure to VP-OEO, a reduction in cell viability begins. In reality, after 12 h of exposure to VP-OEO, 100% of C. albicans Ca2 cells were already PI stained (Fig. 3B2) in mature biofilms, although during biofilm formation, only 50% of cells were stained with PI (Fig. 3A2). The same effect was observed in relation to metabolic activity at 12 h, where mature biofilms showed no metabolic activity (Fig. 3B3), while during biofilm formation, cells ceased to have metabolic activity only after 18 h of exposure to VP-OEO (Fig. 3A3). Since after the absorption and accumulation of FUN-1 in the cell’s cytoplasm, the probe is converted only by metabolically active yeast cells, the fluorescence intensity was significantly higher in *Candida* cells exposed to VP-OEO than in untreated cells (control), corresponding to an SI of >1. Thus, an SI of >1 after treatment indicated a serious impairment of yeast metabolism (51). To confirm the cell morphology and viability of C. glabrata Cg8 and C. albicans Ca2 biofilms (formation and preformed biofilms) after 12 h of contact with VP-OEO, the biofilms were observed by confocal microscopy (Fig. 3C and Fig. 4C). Visualization by live/dead staining showed a reduction in C. albicans Ca2 biofilms after exposure to VP-OEO (Fig. 3C). The control shows green, which means that the 24-h-old and 48-h-old biofilms had developed, and most of the cells were alive. However, biofilms exposed to VP-OEO presented more dead cells (red staining) and a poorly developed and sagging architecture (Fig. 3C) compared to the untreated biofilm.

Regarding the effect of VP-OEO on C. glabrata Cg8, it was found that VP-OEO started to exert an effect after 12 to 14 h of exposure (Fig. 4A1 and Fig. 4B1), relatively later than for C. albicans Ca2 (Fig. 3). In fact, from the microscopy images (Fig. 4C), it seems that 12 h of exposure to VP-OEO did not interfere with the structure of the biofilms; despite this, biofilms had more dead cells (red), for both biofilm formation and mature biofilms. Indeed, there was a total loss of metabolic activity and damage to the cell membrane only after 18 h of exposure to VP-OEO, with 100% of the cells being stained with PI, for both biofilm formation and mature biofilms, although, there was also a great difference in cell viability at 14 h between mature biofilms (50% PI-stained cells) and biofilm formation (only 4% PI-stained cells), with the same trend being observed for C. albicans Ca2 cells (Fig. 3). This trend observed in the two *Candida* species, with VP-OEO having a faster effect on mature biofilms than on the formation stage, contradicts what was expected since mature biofilms present greater resistance. Interestingly, these results are in agreement with those of previous studies in which the impact of essential oil (VP-TTEO) was greater on mature biofilms than on biofilm formation; Fernandes et al. (31) suggested that the mechanism of action of EOs on biofilms can be potentiated by characteristics acquired by mature biofilms.

Based on these findings, two of the mechanisms of action of VP-OEO are interference with the integrity of the *Candida* membrane and interruption of cell metabolism. VP-OEO seems to cause serious primary cellular damage to *Candida* cells once the passage of PI into the cells occurred in a significant way, with this aspect being indicative of the existence of membrane damage/lesions. In fact, after membrane destabilization, cells lose their proper homeostasis, which leads to an organic imbalance that blocks their metabolic functions, and consequently, cells lose functionality (9, 52). As described above, carvacrol, one of the main constituents of OEO (36.97%), may be directly related to the modification of membrane permeability by chemical reactions with amino and hydroxylamine groups of membrane proteins (23, 41–43). Corroborating the results of this research, López et al. (53) previously observed that the inhibition of planktonic cells of C. albicans by exposure to VP-OEO can be attributed largely to carvacrol. Furthermore, Niu et al. (54) found that carvacrol induced reactive oxygen species (ROS) production and mitochondrial dysfunction in C. albicans; still, those authors suggested that carvacrol treatment led to the apoptosis of C. albicans cells independent of ROS production.

**FIG 5:**
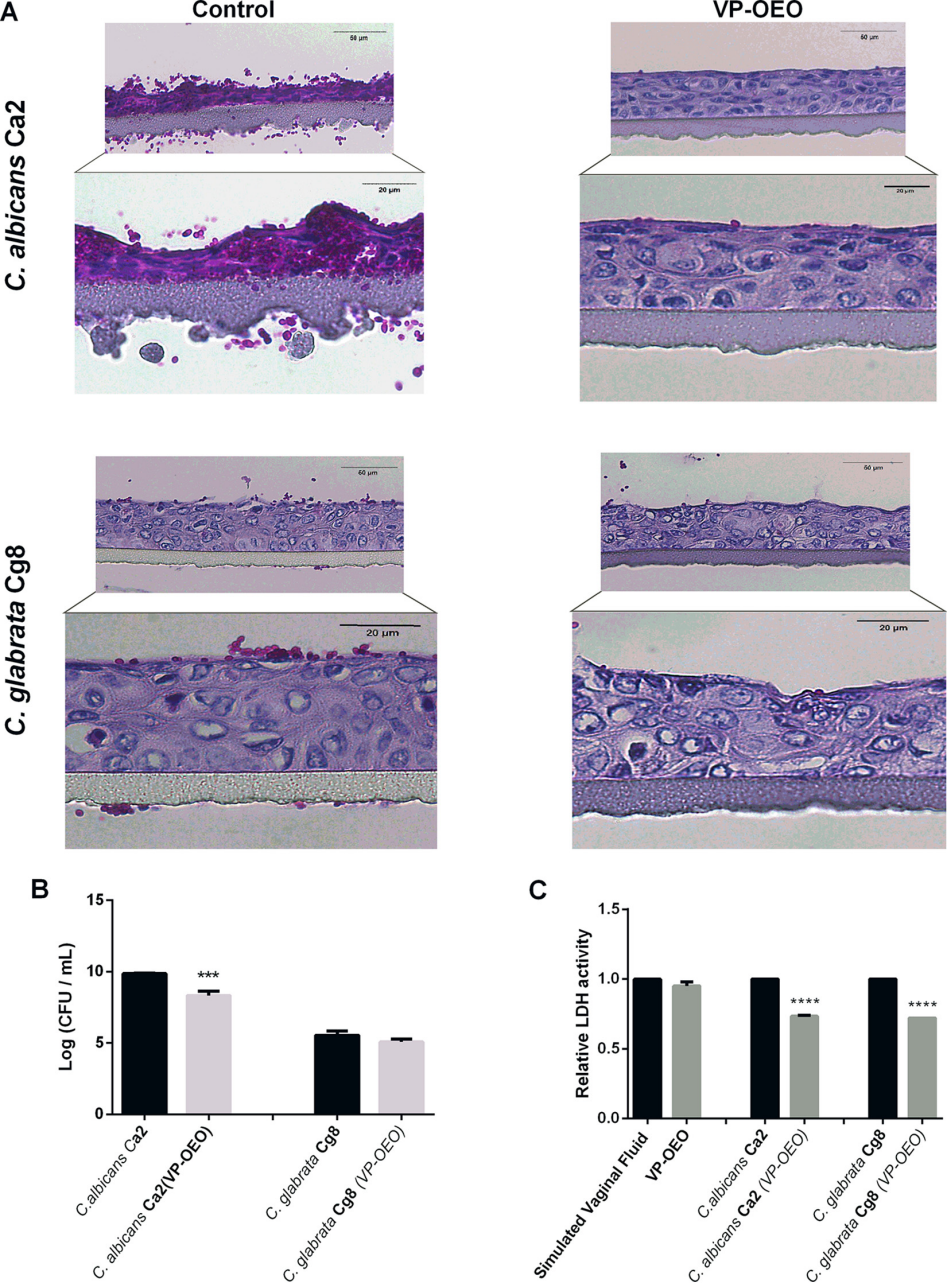
Effect of the vapor-phase of oregano essential oil (VP-OEO) on *Candida* species infection of the reconstituted human vaginal epithelium (RHVE). (A) C. albicans Ca2 and C. glabrata Cg8 infection of the RHVE and effect of VP-OEO after 24 h. (B) C. albicans Ca2 and C. glabrata Cg8 infection of the RHVE after 24 h (log CFU per milliliter). (C) Relative lactate dehydrogenase (LDH) activity measured in the RHVE culture supernatant after 24 h of incubation with the C. albicans Ca2 and C. glabrata Cg8 strains compared to the untreated epithelium (without VP-OEO) and effect of VP-OEO compared to simulated vaginal fluid. Asterisks indicate a statistically significant reduction in biofilm cell cultivability in comparison with that of the respective control (***, *P* < 0.001; ****, *P* < 0.0001).

The reconstituted human vaginal epithelium (RHVE) model has been successfully used as a model for antifungal drug delivery and for studies of the *in vitro* mechanisms of tissue degradation and virulence gene expression after infection with *Candida* species (55). Thus, in the last step of this research, the RHVE was used to mimic vaginal conditions and evaluate the effect of VP-OEO on the attenuation of *Candida* species infection (Fig. 5).

The colonization of the RHVE by *Candida* species was evaluated after 24 h, and the results confirm the effectiveness of VP-OEO against colonization by both *Candida* species (Fig. 5). The greatest effect was observed against C. albicans Ca2, with a reduction in numbers of around 2 logs (*P* < 0.001) in the RHVE exposed to VP-OEO (Fig. 5A and B). Instead, although the effect of VP-OEO on C. glabrata Cg8 was not as pronounced as that on C. albicans Ca2, the cell concentration in the control was also considerably lower, as shown in Fig. 5A.

Lactate dehydrogenase (LDH) is a stable cytosolic enzyme that is released after cell lysis, and the amount of color formed during the LDH assay is proportional to the number of lysed cells (56). Thus, to quantify possible damage to the RHVE induced by VP-OEO, LDH activity levels were measured after 24 h of exposure (Fig. 5C), and the results revealed no significant effects on the RHVE compared to the control (simulated vaginal fluid [SVF]). These results highlight the safe application of the EO and confirm the *in vivo* cytotoxicity results (Fig. 5C). In addition, there was also a significant decrease (*P* < 0.0001) in the amount of LDH released by the RHVE infected by *Candida* species when treated with VP-OEO compared to the untreated RHVE infected with the same strains of C. albicans and C. glabrata. The RHVE model confirms the effectiveness of VP-OEO for the treatment of *Candida* vaginal infections.

The antifungal agents used for *Candida* therapy are often toxic and have low efficacy, particularly against resistant strains of *Candida* species. The replacement of these agents with natural substances is a field to be investigated to reduce the concentration of antifungal agents and increase the effectiveness of treatments. The clinical application of EOs may be discouraged due to their lipophilic character, which makes them potentially toxic when used neat or at high concentrations. However, our study shows that the application of VP-OEO is safe, does not present cytotoxicity, and is active against both *Candida* species (C. albicans and C. glabrata). In fact, our results demonstrated that VP-OEO causes damage to the cell membrane and disrupts the cell metabolism of both *Candida* species studied. Thus, this new EO application approach can be considered a first approach for the development of a complementary or alternative form of antifungal treatment.

## MATERIALS AND METHODS

### Oregano essential oil.

This study evaluated the antifungal activity of oregano (Origanum compactum) EO (Florame, France) with 100% purity. OEO samples were stored at room temperature under dark conditions.

Chromatographic analysis (gas chromatography [GC]) of the OEO was performed by Florame (Saint-Rémy-de-Provence, France) (data not shown). The OEO samples were introduced into the GC system (6890 GC and 5975 mass spectrometry [MS] systems) under the following conditions: a 1-μL injection split of 1/200 was used, an HP5 MS capillary column (30 m/0.25 mm) with a film thickness of 0.25 μm was used, the column temperature was 60°C to 250°C, helium was used as the carrier gas at a constant flow rate of 1.0 mL/min, and the mass range was set at 40 to 450 atomic mass units (amu). The acquisition time was 100 min.

### Oregano essential oil cytotoxicity.

To assess the toxicity of VP-OEO, the Galleria mellonella survival model was used, and the rate of larval survival was determined. For this, 10 G. mellonella larvae were placed into glass petri dishes containing a glass well where the oil was placed (controlled atmosphere with a volume of 1.5 × 10^6^ cm^3^), allowing its diffusion without contact with the larvae. Larvae were stored at 37°C under dark conditions, and larval survival was monitored over 72 h. In addition, the total number of hemocytes present in the hemolymph of the larvae after exposure to VP-OEO was evaluated at 4 h and 72 h, as described previously by Araújo et al. (34). Briefly, 5 larvae sanitized with 70% (vol/vol) ethanol were punctured in the abdomen with a sterile needle, and the hemolymph was collected into a sterile microtube and diluted 10 times in phosphate-buffered saline (PBS). Next, the hemocytes were counted with a Neubauer chamber, and the results were presented as the logarithm of the concentration (log_10_). The control was carried out in the same way, without the larvae being exposed to VP-OEO. All experiments were performed in triplicate in a minimum of three independent assays.

### Microorganisms and culture conditions.

In this study, 13 drug-resistant *Candida* isolates, C. albicans (*n* = 3), C. glabrata (*n* = 8), C. krusei (*n* = 1), and C. guilliermondii (*n* = 1), belonging to the Biofilm Research Group of the Centre o Biological Engineering, were used (57). The clinical isolates were subcultured from a frozen stock (Sabouraud dextrose broth [SDB; Liofilchem] medium with 20% [vol/vol] glycerol at −80°C ± 2°C) onto Sabouraud dextrose agar (SDA; Liofilchem) plates and incubated for 24 h. Prior to testing, a preinoculum was prepared in SDB with colonies from the SDA plates for 18 h at 37°C under agitation (120 rpm). Next, the cellular suspensions were centrifuged and washed twice with PBS (5,000 × *g* for 10 min at 4°C).

For biofilm assays, vaginal *Candida* isolates cells were cultivated in simulated vaginal fluid (SVF). SVF consisted of 58 mM NaCl (Biochem), 18 mM KOH (AppliChem), 2 mM Ca(OH)_2_ (Frilabo), 1.75 mM glycerol (Biochem), 6.7 mM urea (Frilabo), 33 mM glucose (Biochem), and 6.7 g/L yeast nitrogen base (YNB;(Difco)). In addition, natural compounds in the vaginal fluid, such as acetic acid (17 mM; pK_a_, 54.76) and lactic acid (22 mM; pK_a_, 53.85), were added to maintain the pH at 4.2 as described previously by Sosinska et al., Owen and Katz, and Fernandes et al. (58–60).

### Planktonic antimicrobial susceptibilities to oregano essential oil.

The inhibitory effects of direct contact with OEO (DC-OEO) and VP-OEO on the growth of 13 drug-resistant *Candida* isolates were first evaluated by the agar disk diffusion method, as described previously by Tran et al. (61) and Vihanova et al. (62), with some modifications. Briefly, the agar surface was inoculated using a swab dipped in a cell suspension adjusted to 1 × 10^8^ cells/mL. After the inoculum was dried, a sterile filter paper disk (6 mm) (Liofilchem) was impregnated with 25 μL of OEO and directly and indirectly (for vapor-phase action) applied, that is, placed on top of the plates on the cell suspension (direct application) and placed on the cover of the SDA plate, without touching the cell suspension, and incubated inverted (indirect application). Plates with disks without OEO were also included as controls. All plates were then sealed with parafilm and incubated at 37°C, and the diameters of the zones around the disks were measured within 24 h. All experiments were performed in triplicate. The experiments were carried out in glass petri dishes due to the high corrosive power of 100% EOs, according to our unpublished data.

### Effect of the vapor phase of oregano essential oils on biofilms.

The antifungal effects of the VP-OEOs on both biofilm formation and mature biofilms (24 h old) of two fluconazole-resistant *Candida* isolates (C. glabrata Cg8 and C. albicans Ca2) were evaluated. The *Candida* isolate biofilms were developed as described previously by Stepanović et al. (63) and Fernandes et al. (31). After the preinoculum was resuspended and washed twice with PBS, the initial cell concentration was adjusted to 1 × 10^8^ cells/mL in SVF. Next, the cellular suspension was transferred to glass wells inside glass petri plates (1 mL/well) (controlled atmosphere with a volume of 1.5 × 10^6^ cm^3^) and incubated aerobically for 24 h (120 rpm at 37°C). The effect of the VP-OEO was evaluated from two different perspectives: (i) prophylactic treatment, where the therapeutic agent (25 μL of OEOs [100%]) was placed on a sterile blank disk, which was positioned near the wells (the set was kept inside a glass plate), and added at time zero, and (ii) infection treatment, where the therapeutic agent was added to preformed biofilms (24 h).

After 24 h of treatment, the wells were washed with a saline solution, and the resulting biofilm-cell suspensions were then serially diluted in PBS, plated onto SDA plates, and incubated aerobically for 24 h at 37°C. Next, the number of grown colonies was counted. The results were expressed as log CFU per milliliter. The experiment was performed in triplicate with three independent assays.

### Determination of the mechanism of action.

**(i) Biofilm time-dependent killing assay.** After the above-described assays, we decided to perform this part of the study only with C. glabrata Cg8 and C. albicans Ca2 biofilms. The effect of VP-OEO on these species was evaluated by a time-dependent killing assay. This assay was performed according to methods described previously by Sumiyoshi et al. (50), with some modifications. *Candida* biofilms were formed as described above. Inhibition of both biofilm formation and preformed biofilms over time after exposure to VP-OEO (0, 2, 4, 8, 12, 14, 17, 18, and 24 h) was measured. For this, after antifungal treatment and subsequent washing with a saline solution, the suspensions were serially diluted in PBS, plated onto SDA plates, and incubated aerobically (24 h at 37°C). Afterward, the number of grown colonies was counted, and the results were expressed as log reductions in the CFU per milliliter. Each analysis was performed in triplicate.

**(ii) Flow cytometry.** According to the biofilm time-dependent killing assay, preformed and mature biofilms of C. glabrata Cg8 and C. albicans Ca2 after 10, 12, 14, and 18 h of exposure to VP-OEO were analyzed by flow cytometry. Biofilms were formed as described above, and samples of 1 mL were collected after VP-OEO exposure. *Candida* cells killed by treatment with 70% ethanol for 20 min and *Candida* cells without treatment were used as controls. The cells were centrifuged at 4°C for 3 min at 10,000 × *g*, the pellets were resuspended and washed twice with PBS, the suspension concentration was adjusted to 1 × 10^6^ cells/mL, and the cells were counted in a Neubauer chamber, as described previously by Pina-Vaz et al. (64). Two fluorescent probes were used, propidium iodide (PI) and FUN-1 {2-chloro-4-[2,3-dihydro-3-methyl-(benzo-1,3-thiazol-2-yl)-methylidene]phenylquinolinium iodide}. To evaluate cell membrane integrity, cell suspensions were incubated for 30 min in the presence of PI (1.0 μg/mL) (Sigma-Aldrich, Munich, Germany) in the dark at room temperature. To assess the metabolic activity, the cellular suspensions were centrifuged, and the pellets were resuspended in sterilized H_2_O supplemented with 2% glucose and incubated in the presence of 0.5 μM FUN-1 (Invitrogen Molecular Probes, OR, USA) for 30 min with protection from light. For each isolate, nonstained cells (autofluorescence) and the fluorescence of nontreated and dead controls were also evaluated. The samples were analyzed with a FACSCalibur cytometer (BD Biosciences, Sydney, Australia) equipped with 3 photomultipliers (PMTs), standard filters, and a 15-mW, 488-nm argon laser using CellQuest Pro software (version 4.0.2). The cell scattergram (forward scatter [FS] and side scatter [SS]) and the intensity of fluorescence at fluorescent channel FL1 (green fluorescence, 530 nm), FL2 (yellow-green fluorescence, 575 nm), and FL3 (red fluorescence, 630 nm) were logged using a logarithmic scale.

PI results are expressed as a percentage of cells showing high fluorescence in FL3, and FUN-1 results are expressed as the staining index (SI), defined as the ratio between the mean fluorescence of the treated cell suspensions (VP-OEO) and the value corresponding to the control cells (no exposure to VP-OEO), in FL2 (64). Each assay was performed in triplicate.

**(iii) Confocal laser scanning microscopy analysis.** In order to evaluate the cell morphology and viability of C. glabrata Cg8 and C. albicans Ca2 biofilms (biofilm formation and preformed biofilms) after 12 h of exposure to VP-OEO, the Live/Dead BacLight bacterial viability kit (Molecular Probes, Leiden, The Netherlands) was employed. Briefly, biofilms were formed on glass coupons (inserted into the glass wells, as described above) after 12 h of exposure to VP-OEO, for both biofilm formation and preformed biofilms, and the coupons were washed with 0.85% NaCl and then stained for 15 min in the dark with a mixture of SYTO-9 (3 μL/mL) and PI (3 μL/mL). Samples were observed using an Olympus (Tokyo, Japan) BX61 model FluoView 1000 confocal scanning laser microscope. The combination of optical filters consisted of a laser excitation line at 488 nm, emission filters BA 505 to 540 nm (green fluorescence) with an excitation line at 559 nm, and emission filters BA 575 to 675 nm (red fluorescence). Images were acquired and analyzed with the FV10-Ver4.1.1.5 program (Olympus). The assay was repeated two independent times with two technical replicates.

### Effect of oregano essential oil on *Candida* species infection of a reconstituted human vaginal epithelium.

To mimic human vaginal conditions, a commercially reconstituted human vaginal epithelium (RHVE) (0.5 cm^2^; SkinEthic Laboratories) was infected with *Candida* species (C. glabrata Cg8 and C. albicans Ca2), and the effect of VP-OEO on vaginal candidiasis attenuation was evaluated. For this, epithelia were infected with a cell suspension of C. glabrata and C. albicans (initial concentration adjusted to 1 × 10^8^ cells/mL in SVF). Epithelia devoid of *Candida* cells but with SVF exposed and unexposed to VP-OEO were used as controls. All epithelia were incubated for 24 h (120 rpm at 37°C). After incubation, the tissues were washed once with PBS to remove nonadherent *Candida* cells. Epithelia were then bisected, with one half being used for histological analysis and the other half being used for molecular studies (55).

**(i) Microscopic observation.** RHVE tissue for microscopic analysis was fixed in 2% (vol/vol) paraformaldehyde, stored at room temperature, and embedded in paraffin wax using standard histological techniques. RHVE sections (20 μm) were cut, placed onto HistoBond^+^-coated microscope slides, and dewaxed by processing through xylene, followed by immersion in ethanol and then in water. The prepared sections were then stained using the periodic acid-Schiff method for keratinocyte and *Candida* cell staining.

After histological processing, samples were analyzed in bright field using an Olympus BX51 epifluorescence microscope coupled with a DP72 digital camera (Olympus Portugal SA, Portugal). All tissue images were acquired using Olympus Cell-B software.

**(ii) Quantification of *Candida* cells in an RHVE.** DNA present in the tissues was extracted using a DNA extraction kit (DNeasy blood and tissue kit; Qiagen). *Candida* cells were quantified using real-time PCR by employing a CFX96 real-time PCR system (Bio-Rad, Berkeley, CA, USA). Each reaction mixture consisted of 10 μL of a working concentration of SsoFast EvaGreen supermix, 0.2 μL of each primer (forward primer 5′-GAGCGTCGTTTCTCCCTCAAACCGCTGG-3′ and reverse primer 5′-GGTGGACGTTACCGCCGCAAGCAATGTT-3′ for C. albicans and forward primer 5′-ATTTGCATGCGCTTGCCCACGAATCC-3′ and reverse primer 5′-ACGTCTGATCCAATCAATGGCTGGTGA-3′ for C. glabrata), and 4 μL of DNA, in a final reaction mixture volume of 20 μL. Negative controls were performed using a reaction mixture with nuclease-free water replacing the DNA template. PCR cycling conditions consisted of an initial denaturation step at 94°C for 3 min followed by 40 cycles of denaturation at 95°C for 10 s and primer annealing at 55°C for 30 s. For each *Candida* isolate, calibration curves (*C_T_* [threshold cycle] versus log cells) were constructed using the same PCR protocol as the one described above, from serial dilutions of *Candida* cell concentrations, as described previously by Alves et al. (55).

**(iii) Lactate dehydrogenase assay.** The release of lactate dehydrogenase (LDH) from the RHVE into the culture medium was used as a measure of epithelial cell damage using the CytoTox-ONE homogeneous membrane integrity assay kit (Promega). The LDH released during infection and VP-OEO treatment for the two *Candida* strains was expressed as LDH activity relative to the values for untreated C. albicans Ca2 and C. glabrata Cg8. In addition, the effect of VP-OEO on tissue, expressed as the LDH activity relative to that in tissue with SVF (control), was also evaluated. LDH activity was analyzed in a spectrophotometer (FLUOstar Optima; BMG Labtech, Ortenberg, Germany) at a 560-nm excitation wavelength and a 590-nm emission wavelength. All experiments were performed in triplicate.

### Statistical analysis.

The results were statistically analyzed using the Prism software package (version 8.01; GraphPad Software). One-way analysis of variance (ANOVA) was performed, and means were compared by applying Tukey’s multiple-comparison test. The statistical analyses performed were considered significant when the *P* value was <0.05.

### Data availability.

All data generated or analyzed during this study are included in this article.
